# Toxic elements and fatty acid composition in the freshwater fish family Cyprinidae (Rafinesque 1815): balancing nutritional benefits and health risks

**DOI:** 10.1007/s10661-025-14112-4

**Published:** 2025-05-26

**Authors:** Anton Kovacik, Marek Helczman, Julius Arvay, Tomas Jambor, Eva Kovacikova

**Affiliations:** 1 Institute of Applied Biology, Faculty of Biotechnology and Food Sciences, Slovak University of Agriculture in Nitra, Tr. A. Hlinku 2, 949 76 Nitra, Slovak Republic; 2 Institute of Food Sciences, Faculty of Biotechnology and Food Sciences, Slovak University of Agriculture in Nitra, Tr. A. Hlinku 2, 949 76 Nitra, Slovak Republic; 3 Institute of Nutrition and Genomics, Faculty of Agrobiology and Food Resources, Slovak University of Agriculture in Nitra, Tr. A. Hlinku 2, 949 76 Nitra, Slovak Republic

**Keywords:** Heavy metal, Metalloid, Fatty acid, Toxicity, Freshwater fish, Cyprinidae

## Abstract

The aim of this study was to assess the toxicity of heavy metals/metalloids, including arsenic, cadmium, lead, and mercury accumulated in the muscle of commonly consumed fish from the Cyprinidae. We discussed the importance of fatty acids in the human diet and investigated their profile in the muscle of different fish species. Additionally, our goal was to evaluate the benefits of fish consumption in relation to its risks, not only by considering the advantages of fatty acids and the drawbacks of heavy metal toxicity but also by examining how these pollutants may alter the fatty acid profile in fish muscle, potentially reducing the quality of their nutritional benefits. We categorized these fatty acids based on their proportions in total lipids into muscle tissue of the SFA (saturated fatty acids), MUFA (monounsaturated fatty acids), and PUFA (polyunsaturated fatty acids) groups. Subsequently, we have described the toxic effects of selected elements on human health, reviewing that investigated exposure levels of these toxic elements in fish muscle and the safety of consumption through risk assessment tools such as total hazard quotient (THQ) and carcinogenic risk (CR) calculations. In the final section we focused on lipid metabolism, which is significantly affected by exposure to toxic elements. We searched for a possible relationship between the presence of toxic elements and changes in the fatty acid profile of fish muscle. The knowledge from other studies led us to the possibility of a lower PUFA content due to the damage of double bonds and the subsequent degradation of these fatty acids. Total fatty acid profile is a crucial factor in evaluating health risks and serve as an important indicator of fish meat quality. On the other hand, it can serve as a potential indicator of environmental contamination by these toxicants.

## Introduction

Currently, pollution of the freshwater environment by non-essential toxic elements is a serious burden not only for fish and other aquatic organisms (Javed & Usmani, 2019; Kolarova & Napiórkowski, 2021; Pastorino et al., 2021; Reis et al., 2019), but also for humans as the final consumers in the food chain (Ali & Khan, 2018; Campbell & Gailer, 2016; Copat et al., 2012). Toxic metals such as cadmium (Cd), lead (Pb), and mercury (Hg), or metalloid arsenic (As), in addition to their natural exposure, enter the environment primarily through anthropogenic activities, whether through industrial production or agriculture (Acosta-Pachón et al., 2024; Andreji et al., 2006; Evers et al., 2024; Gomes et al., 2024; Kovacik et al., 2019; Mushtaq et al., 2020). Fish are constantly exposed to the external environment and to these elements from the water, thereby making them an ideal model for ecotoxicology research (Dai et al., 2014; Helczman et al., 2024; Shahjahan et al., 2022). Studies show that the bioaccumulation of these toxic elements in the fish body is an important indicator of environmental pollution (Ali et al., 2019; De Paula Gutiérrez & Agudelo, 2020; Van Der Oost et al., 2003; Zaghloul et al., 2020). The highest accumulation of toxic elements in tissues has been observed mainly in organs such as gills, liver and kidneys, as these are detoxifying and water filtering organs (Deb & Fukushima, 1999; Kumar et al., 2024; Milošković et al., 2022; Squadrone et al., 2013). However, bioaccumulation in muscle tissue cannot be underestimated either (S. Hussain et al., 2021; Naeem et al., 2021; Shah et al., 2020; Shahjahan et al., 2022).

Fish muscle constitutes the most important tissue for human consumption. As highly regarded food source, fish meat is considered to be a valuable due to its nutritional value (Ahmed et al., 2022; Modzelewska-Kapituła et al., 2017; Venugopal & Shahidi, 1996). Fish exhibits an interesting nutritional profile characterized by its content of high-quality fatty acids, notably long-chain omega-3 fatty acids (ω−3) (Arab-Tehrany et al., 2012; Özden et al., 2020). These components are an integral part of various dietary recommendations approved by experts and underline the importance of fish as an essential element in a balanced diet (J. Chen et al., 2022; Heileson et al., 2023; Mendivil, 2021; Tacon et al., 2020). However, toxic elements accumulated in fish muscle can pose a significant risk to human health when consumed (Bosch et al., 2016; Kormoker et al., 2021; Kovacik et al., 2024; Phillips et al., 2014; Wolff et al., 2016). Earlier research has shown that these elements, at high concentrations, have carcinogenic, neurodegenerative and other cytotoxic effects (Bhattacharjee et al., 2013; Budi et al., 2024; Dasharathy et al., 2022; Tchounwou et al., 2012). For this reason, there are many regulations setting maximum permitted levels of these elements in drinking water and in various foods, including fish meat (Wong et al., 2022). Based on these regulations, it is possible to calculate the total hazard quotient (THQ) and carcinogenic risk (CR) to determine the potential risk of consumption of such contaminated fish (Fathabad et al., 2021; Jovanović et al., 2017; Kebede & Geleta, 2023; Kovacik et al., 2024; X. Wang et al., 2020). These calculations provide information that can help determine the safety, and to recommend (or not) the consumption of the fish evaluated (Fakhri et al., 2021; Varol & Sünbül, 2018). However, while these calculations assess safety, they do not include possible changes in lipid metabolism that could affect the quality of the meat. Toxic elements affect biochemical and oxidative stress parameters in the body (Kovacik et al., 2023; Lushchak, 2016; Sevcikova et al., 2011; Shaikh et al., 1999), which in consequence leads to lipid peroxidation (Lawton & Donaldson, 1991) and many other processes. It can often cause the degradation of certain fatty acids that are of high importance for the fish body, but also for their presence in the human diet (Azevedo et al., 2009; Das et al., 2018). If, as a result of these changes, there were statistically significant decreases in the overall profile of these fatty acids, then the benefit of consuming fish meat may be lower, and the risk/benefit ratio would have changed (Bosch et al., 2016; Domingo et al., 2007; Özden et al., 2020).

The aim of this review is to observe the inter-species fatty acid composition in the overall fatty acid profile of fishes from family Cyprinidae, and to monitor the risk/benefit ratio in the studies carried out with exposure to toxic elements at different concentrations. We also hope to identify correlations between selected toxic elements concentrations and fatty acid composition, and to clarify the relevance of lipid metabolism for ecotoxicological studies in this field. We hypothesize that changes in the fatty acid profile could be an indicator not only of the quality of fish meat, but also an indicator of toxic elements pollution in the freshwater environment.

**Fig. 1:**
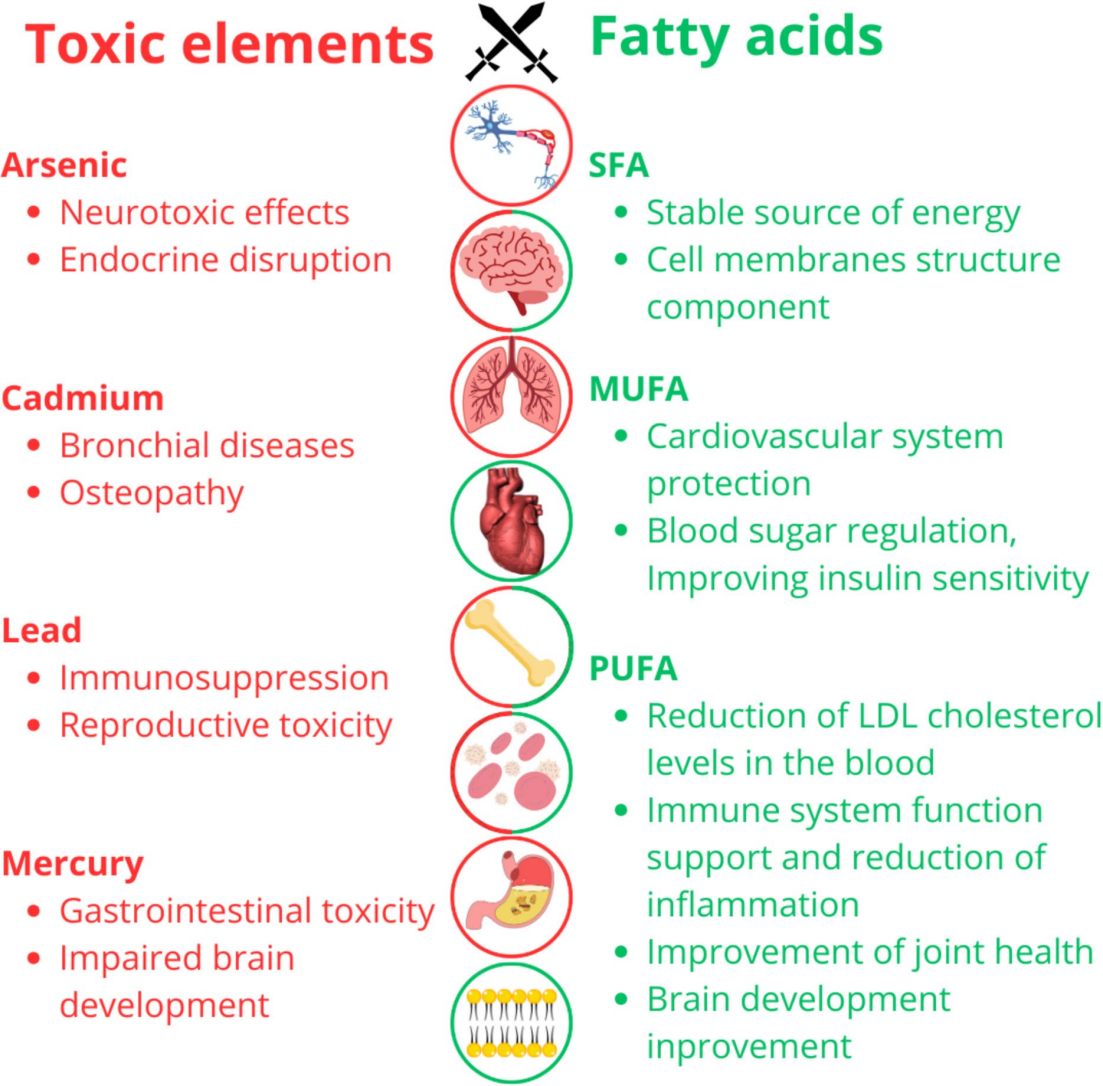
Illustration of the impact of selected toxic elements and fatty acids on human health. Scheme was created by authors using Canva (https://www.canva.com/)

This review has been designed as a compilation of previous studies investigating the beneficial effects of fish meat consumption in terms of fatty acid benefits and the risks associated with such consumption due to the accumulation of toxic elements (As, Cd, Pb, Hg) in fish muscle, considering the possible effects of these elements on fatty acid degradation (Fig. 1). A search of scientific databases Web of Science, PubMed, and Google Scholar was performed using the keywords"freshwater fish","Cyprinidae","bioaccumulation","toxic elements"or"heavy metals","Arsenic","Lead","Cadmium","Mercury","fatty acids","health/risk assessment","lipid peroxidation"for the period from 2010 to 2024. These key terms were searched in combinations. Subsequently, studies involving marine environments, marine fish or fish species other than Cyprinidae, articles investigating the combined effects of elements accumulation with another pollutant, and studies that did not investigate the toxic elements that were the subject of our study were excluded from the search.

## Freshwater fish as a source of important fatty acids in human nutrition

Fish are rich in many essential nutrients. They provide high-quality, easily digestible and usable proteins that are an excellent source of essential amino acids (Bhowmik et al., 2022; Hasselberg et al., 2020; Islam et al., 2021; Mohammadi et al., 2023; Mohanty et al., 2019). Additionally, fish oil is rich in lipids, the types and amount of which are most closely monitored. Among these, polyunsaturated fatty acids (PUFA), such as linoleic acid and linolenic acid, which are classified as essential for human nutrition (Galindo et al., 2021; Sun et al., 2020).

Studies analysing composition of fish clearly demonstrated that the content and amount of individual nutrients depend on various factors, including the water environment (fresh, salty, warm or cold) where the fish are found. Biodiversity within fish species also contribute to variations in nutrient composition of fish (Bhowmik et al., 2022; Hasselberg et al., 2020; Mielcarek et al., 2022; Sroy et al., 2021). Factors as fish species, age, sex, body size, seasonal changes, feeding and environmental conditions can also influence the fatty acids composition.

Omega-6 (ω−6) and omega-3 (ω−3) polyunsaturated fatty acids are important metabolic precursors of long-chain (C 20–24) PUFAs (LC-PUFAs), such as arachidonic acid (20:4n-6, ARA), eicosapentaenoic acid (20:5n-3, EPA), and docosahexaenoic (22:6n-3, DHA) acid, mostly found in fatty fish (Ahmed et al., 2022; Galindo et al., 2021; Sun et al., 2020; Van Hecke et al., 2019). For optimal dietary intake of these essential PUFAs, 4–5:1 is the recommended ratio of ω−6:ω−3 fatty acids. This ratio impacts human health positively (He, 2009; Li et al., 2021; Mariamenatu & Abdu, 2021). Recently, an increased intake of ω−6 fatty acids has been observed, especially in countries following western type of diet. At the same time, ω−3 fatty intake acids remain insufficient. Regular consumption of fish is one of the possible solutions to help maintain a proper balance between ω−6 and ω−3 fatty acids in the diet (Mariamenatu & Abdu, 2021; Molendi-Coste et al., 2011; Weaver et al., 2008). Table 1 shows the differences in fatty acid content among different fish species from various locations.

**Table 1:** Studies that investigated fatty acid composition of fish (family Cyprinidae) muscle tissue

Species	TL (Total Lipids) g/100 g of wet weight	PUFA % of TL	MUFA % of TL	SFA % of TL	Reference
*Cyprinus carpio*	12.15 ± 0.45	32.58 ± 1.58	21.05 ± 1.30	46.37 ± 2.10	(Stancheva et al., 2014)
*Cyprinus carpio (Scaly carp)*	-	35.08 ± 1.81	33.85 ± 1.11	31.07 ± 1.24	(Aydın et al., 2022)
*Cyprinus carpio (Mirror carp)*		35.72 ± 1.89	32.68 ± 1.29	31.43 ± 1.57	
*Squalius cephalus (spring)*	-	40.82 ± 0.16	26.23 ± 0.37	32.96 ± 0.19	(Görgün & Akpınar, 2019)
*Squalius cephalus (summer)*		40.99 ± 0.10	25.42 ± 0.11	33.58 ± 0.25	
*Squalius cephalus (autumn)*		39.65 ± 0.11	21.32 ± 0.10	39.06 ± 0.22	
*Squalius cephalus (winter)*		36.51 ± 0.13	33.74 ± 0.12	29.75 ± 0.13	
*Rutilus rutilus*	-	27.189	36.499	36.311	(Jovičić et al., 2023)
*Blicca bjoerkna*		5,873	43.703	50.424	
*Abramis brama*	2.17 ± 0.19	37.8 ± 11.4	37.9 ± 10.6	25.7 ± 5.51	(Khalili Tilami et al., 2018)
*Carassius gibelio*	1.94 ± 1.13	41.33 ± 6.10	35.59 ± 11.11	22.88 ± 4.41	
*Chondrostoma nasus*	4.04 ± 0.81	27.80 ± 5.91	42.17 ± 5.53	22.24 ± 3.65	
*Squalius cephalus*	3.49 ± 0.53	34.71 ± 2.80	40.62 ± 3.03	23.23 ± 1.26	
*Aspius aspius*	2.78 ± 0.11	24.60 ± 0.3	47.69 ± 0.37	27.99 ± 0.29	(Ljubojevic et al., 2013)
*Abramis brama*	3.24 ± 0.15	17.07 ± 0.27	56.09 ± 0.34	27.27 ± 0.24	
*Barbus barbus*	7.78 ± 0.15	26.31 ± 0.27	45.27 ± 0.3	28.63 ± 0.27	
*Cyprinus carpio*	7.13 ± 0.1	19.7 ± 0.49	52.94 ± 0.18	27.59 ± 0.19	
*Cyprinus carpio*	6.85 ± 0.14	10.95 ± 0.09	64.34 ± 0.06	24.23 ± 0.06	(Ćirković et al., 2012)
*Hypophthalmichthys molitrix*	4.07 ± 0.05	24.23 ± 0.18	39.04 ± 0.08	34.05 ± 0.08	
*Ctenopharyngodon idella*	6.39 ± 0.24	18.26 ± 0.04	51.29 ± 0.08	29.03 ± 0.09	
*Tinca tinca*	5.78 ± 0.11	22.08 ± 0.43	34.97 ± 0.17	36.36 ± 0.33	
*Ctenopharyngodon idella* *(in summer season)*	-	24.21 ± 6.20	35.30 ± 13.90	40.49 ± 8.22	(Kovacik et al., 2024)
*Ctenopharyngodon idella(in autumn season)*		23.38 ± 4.48	33.62 ± 8.77	42.99 ± 6.40	
*Tor putitora*	-	18.36 ± 0.37	28.06 ± 0.74	52.97 ± 0.10	(Sarma et al., 2013)
*Neolissochilus hexagonolepis*		31.2 ± 0.10	23.95 ± 0.03	44.29 ± 0.20	
*Cyprinus carpio*		23.72 ± 0.29	31.00 ± 0.37	46.19 ± 0.27	
*Carassius gibelio*					
*Summer season*	2.01 ± 0.07	25.02 ± 0.07	41.55 ± 0.15	30.35 ± 0.06	(Dagtekin et al., 2018)
*Autumn season*	2.39 ± 0.24	24.65 ± 0.22	41.28 ± 0.20	29.4 ± 0.09	
*Spring season*	3.49 ± 0.66	21.52 ± 0.28	44.82 ± 0.34	30.27 ± 0.16	
*Rutilus rutilus (location 1)*	-	36.31	36.50	26.92	(Jovičić et al., 2024)
*Abramis brama (location 1)*		50.42	43.70	5.87	
*Rutilus rutilus (location 2)*		45.12	33.56	21.32	
*Abramis brama (location 2)*		51.79	42.89	5.32	

Studies investigating the relationship between fish-derived PUFAs consumption and human health indicate several potential benefits. Among them, protective effect against cardiometabolic (Ateya et al., 2017; Fujii et al., 2021; Harris et al., 2018; He, 2009; Liddle et al., 2020; Mozaffarian & Wu, 2011; Russo, 2009; Weichselbaum et al., 2013) and neurodegenerative diseases (Galindo et al., 2021; Liddle et al., 2020). Mostly, research on the impact of PUFAs on human health focuses on their role in secondary prevention. In most cases, the patients are often given supplements containing ω−3 fatty acids in different amounts and periods. Such therapeutic approach has a positive impact on disease progression and improves biochemical parameters, potentially helping to prevent premature deaths from cardiovascular diseases (Abdelhamid et al., 2018; Hammad et al., 2016; Harris et al., 2018; Mozaffarian & Wu, 2011; Russo, 2009).

The American Heart Association recommends consuming fish 2–3 times a week as a primary prevention strategy against civilization diseases, which equates to an amount of 250 mg of EPA + DHA per day (Mohanty et al., 2019; Rimm et al., 2018; Skulas-Ray et al., 2019). A crucial factor in assessing the health benefits of fish consumption is the method of their technological and heat treatment. It is recommended to avoid frying and processed fish products (Galindo et al., 2021; He, 2009; Vianna et al., 2020).

Fish consumption varies across individual regions of the world, with notable differences between sea and freshwater fish intake (Mielcarek et al., 2022). Fish are affordable sources of animal proteins and have a high biological value (Jabeen & Chaudhry, 2011; Mohanty et al., 2019). Their low cost and high nutritional value make this food source an ideal solution for dietary issues in developing countries, where nutritional deficiencies from malnutrition are a major health problem. On the other hand, developed countries are experiencing an increase in chronic civilization diseases caused by consumption of nutritionally poor food and excess energy intake (Bhowmik et al., 2022; Hasselberg et al., 2020; Mohanty et al., 2019; Vianna et al., 2020).

Lipids and individual fatty acids are responsible for the permeability, fluidity and integrity of biological cell membranes (Banfalvi, 2016; Santos & Preta, 2018). They are precursors of bioactive and signalling molecules, responsible for regulation of biochemical reactions in cells (Bergé & Barnathan, 2005; Calder, 2015). Additionally, they also play a role as ligands for nuclear receptors and provide energy for many biological processes (as adenosine triphosphate—ATP). Lipids are important for proper growth, reproduction, osmoregulation and immune response processes, and are also involved in biochemical processes including signal transduction and metabolic homeostasis (Ferain et al., 2021; Fokina et al., 2020; Fonseca et al., 2022; Glatz & Luiken, 2015). Eicosapentaenoic acid is a precursor of eicosanoids, which have a wide spectrum of physiological effects and play roles in immune and inflammatory responses, nervous system functions, and reproduction (Narayan et al., 2006; Poorani et al., 2016). Eicosanoids also help the organism to adapt to environmental stressors (Fokina et al., 2020).

## Characteristics of selected toxic microelements

### Arsenic

Arsenic naturally occurs in sulphide ores (Chung et al., 2014). Historically, it has been used in pesticides and wood preservatives, or as a decolorizing agent in the production of glass and enamels. Nowadays, it is still utilized to remove sulphur from industrial gases. Its physical properties such as hardness and heat conductivity, make it a valuable additive in the production of various alloys.

Furthermore, in crystalline forms such as artificial gallium arsenide and indium arsenide, it has become a valuable material for space research applications (Fowler et al., 2022; Su et al., 2023).

Arsenic concentrations are generally low in most foods; however, higher levels are often found in fish and seafood due to the widespread presence of arsenic in water. The gastrointestinal tract is responsible for the majority of this metalloid absorption, with about 90% of the ingested dose being absorbed (Fowler et al., 2022; Pomroy et al., 1980; Y. Zheng et al., 2002). The arsenic toxicity to humans has been recognized since ancient times. In contrast, animals are less sensitive to the toxic effects of arsenic, likely due to differences in absorption and methylation processes.

Acute and subacute arsenic toxicity is initially manifested by gastrointestinal issues such as nausea, vomiting, thirst, heartburn, and abdominal pain. Other symptoms can include disorientation, muscle cramps, haematuria, and blood in the stool. Chronic symptoms depend on the dose and time of exposure and may include hyperkeratosis, peripheral neuropathy, anaemia, anorexia, hypertension, type 2 diabetes mellitus, and an increased risk of cancer (Hong et al., 2014; Naujokas et al., 2013; Ozturk et al., 2022; Ratnaike, 2003).

A study carried out by (X. Wang et al., 2015) on rats indicates that arsenic interferes with lipid and carnitine metabolism. The researchers observed an increase in ceramides and lysophosphatidylcholines (lysoPC) in the blood after arsenic exposure, which may be related to changes in the muscle cells lipid membranes. Additionally, research by (Ceja-Galicia et al., 2017) demonstrates that arsenic can negatively affect lipogenesis, lipolysis, and subsequent lipid deposition in adipocytes. Furthermore, arsenic exposure causes muscle weakness, atrophy and muscle loss (Ambrosio et al., 2014).

### Cadmium

Cadmium is a toxic metal classified as a sulphide ore, closely resembling zinc in chemical structure and is often naturally found alongside lead in ores from where it is mined (Nordberg et al., 2022). While its industrial use was extensive in the past, currently there are fewer toxic alternatives for its properties. In some countries, it is still used to protect metallic materials from corrosion. Some of its compounds are employed in the plastics and dyes production (Hayat et al., 2019), but its primary applications today are in batteries and solar panels (Basol & McCandless, 2014). Cadmium enters the environment through anthropogenic activities mainly (Ankush et al., 2024).

Human absorption of Cd by ingestion is higher (4–7%) compared to animal absorption (1–6%). Low intake of protein, fibre, iron, zinc or calcium may lead to increased absorption of Cd (Nordberg et al., 2022). Over a lifetime, accumulation of cadmium in muscles is increasing. Animal and human studies have clearly demonstrated a multiplicative effect when organisms are simultaneously exposed to combined Cd and As concentrations. Kidneys are the most affected organs (Nordberg et al., 2022; Sanders et al., 2019).

Acute cadmium toxicity is characterized by nausea, vomiting and abdominal pain, primarily affecting kidney function and reducing ability to filtrate blood. Symptoms of chronic toxicity from prolonged exposure can include osteoporosis, lung damage, and other kidney diseases. Cadmium is regarded as a carcinogenic substance, chronic exposure often leads to lung and prostate cancer, mainly due to genetic mutations and DNA damage. Additionally, cadmium weakens the immune system and increases the body's vulnerability to other diseases (Charkiewicz et al., 2023; Genchi et al., 2020).

Cadmium intoxication is associated with altered lipid metabolism. Fokina et al. (2020) in their study conducted on the freshwater mussel *Anodonta cygnea,* it was observed that after the initial day of Cd exposure there was a decrease in linoleic acid content and increase in lipid peroxidation. Interestingly, on the third day of exposure, linoleic acid values were increased along with arachidonic acid values, accompanied by increased catalase activity. Additional studies performed on broiler chickens (Zoidis et al., 2019), blue mussels (*Mytilus edulis L*.) (Fokina et al., 2013), rats (Larregle et al., 2008; Shaikh et al., 1999), and *Chlorella vulgaris* (Chia et al., 2013) suggest that cadmium has a significant effect on the fatty acid profile, especially PUFAs. Cadmium appears to promote lipid metabolism, leading to increased formation of triglyceride, arachidonic acid and other fatty acids from the PUFA category. These metabolic changes may elevate the risk of liver diseases (e.g., fatty liver) and other metabolic disorders.

### Lead

Lead is the most well-studied environmental toxicant, with China being the largest producer, followed by Australia and the USA. Its most common applications at present are in batteries and electrical components, particularly backup power supplies (O’Connor et al., 2018; Tian et al., 2017). In some countries, it is still a key component of paints for both interior and exterior use (O’Connor et al., 2018). Lead is protective from corrosion, making it a valuable substance for various metal structures such as bridges and ships. Additionally, it can be found in water pipes, continuously leaching into the aquatic environment and causing toxic contamination of drinking water and the water ecosystem. This contamination represents a risk to aquatic organisms, leading to bioaccumulation in their bodies (Bergdahl & Skerfving, 2022; Demayo et al., 1982).

In 2020, the human burden of lead was up to 100 times higher than in prehistoric times, although this represents a good step forward from the 1990 s (1000 times higher burden), which is mainly due to regulations of its use (Patterson et al., 1991). One of the primary sources of exposure is food consumption, followed by drinking water. Based on studies, it is estimated that, on average, 15–20% of stable lead and approximately 8% of lead salts ingested into the gastrointestinal tract are subsequently absorbed by the body (Bergdahl & Skerfving, 2022).

Acute lead intoxication is characterized by symptoms such as abdominal pain, cramps, nausea, and vomiting, and in severe cases, seizures or coma. Chronic exposure to lead has serious effects on the nervous system, leading to memory impairments, behavioural and cognitive issues. Additionally, it also damages the kidneys and reproductive system. Early symptoms of chronic exposure are often barely recognizable and may go unnoticed (Shukla et al., 2018; Tsai et al., 2017).

Research shows that lead induces oxidative stress, leading to higher amounts of reactive oxygen species (ROS) and insufficient antioxidant substances in the body. This results in DNA and protein damage, as well as lipid peroxidation. Previous studies suggest that Pb significantly affects the metabolism of fatty acids, promoting their peroxidation in the body (Imsilp et al., 2024; Knowles et al., 1998; Lawton & Donaldson, 1991; Mateo et al., 2003). In these studies, a positive correlation was observed between elevated Pb levels and higher concentrations of arachidonic acid (20:4 n-6), along with an enhanced effect of Pb on the fatty acid elongation. Increasing Pb levels in the body have been found to raise the PUFAs, which may significantly affect metabolism and membrane stability, potentially leading to further inflammatory reactions.

### Mercury

Mercury occurs naturally in the form of sulphides, with cinnabar, a red sulphide, being the most known source.. Mercury enters the environment mainly through anthropogenic activities, such as combustion of fossil fuels and industrial processes (Árvay et al., 2022; Demková et al., 2024; Fowler & Zalups, 2022; Gedig et al., 2023). It is widely used in the pharmaceutical industry, in plastics and paints, and also has applications as germicide and fungicide because of the cytotoxic properties (Langan et al., 1987; Pacyna et al., 2010; Veiga et al., 2014). The production of alkylmercury compounds is now prohibited in most countries.

Mercury is abundant in the food chain of aquatic animals due to its accumulation in the sediment of water. The highest levels have been observed in predatory fish (Da Silva et al., 2020; N. Zheng et al., 2019). From the aquatic environment, mercury is further distributed through animals feeding on aquatic organisms. One of the factors influencing methylmercury levels in fish is pH; a positive correlation has been observed between acidification of aquatic ecosystems and mercury levels in aquatic organisms (Fowler & Zalups, 2022).

Mercury binds to tiol groups of proteins, interfering with cell signalling and enzyme functions (Ajsuvakova et al., 2020). Within the central nervous system, it can induce oxidative stress and damage cell membranes. In case of acute poisoning, respiratory problems (such as coughing or pneumonia), headaches, vomiting and kidney damage may occur. More severe cases can result in neurological damage. Long-term exposure leads to bioaccumulation, especially in brain tissue, resulting in neurological disorders, loss of coordination, cognitive impairment, and issues with sensory perception and memory. The danger is even higher for pregnant women, as mercury exposure may lead to fetal harm (Ekino et al., 2007; Ozuah, 2000; Rice et al., 2014).

Several previous studies have suggested that the fatty acid profile of animals may serve as a biomarker of environmental pollution. For instance, a study monitoring changes in the fatty acid profile of the sea slug (*Gibbula umbilicalis*) after exposure to mercury discovered that such exposure alters fatty acid metabolism (Silva et al., 2017). Alterations in 5 fatty acids (palmitic, eicosapentaenoic, eicosatrienoic, arachidonic, and docosahexaenoic) were identified and evaluated as a suitable biomarker for future research.

In another study (Signa et al., 2015), mussels (*Mytilus galloprovincialis*) were transferred from a clean, uncontaminated environment to an environment containing toxic elements, including mercury and other pollutants. The results demonstrated that lipid peroxidation and changes in the fatty acid profile occured, particularly a decrease in PUFA content, following the environmental change.

Additionally, research on *Holothuria forskali* revealed that exposure of mercury to led to modifications in the fatty acid composition, characterized by an elevation of SFA and a decrease of MUFA and PUFA in the overall fatty acids profile (Rabeh et al., 2021). The most significant effects were observed at the lowest concentration tested.

A study investigating the effects of mercury and cadmium exposure in *Sparus aurata* monitored changes in the fatty acid profile alongside bioaccumulation in different tissues (Maria et al., 2022). At the biochemical level, authors observed an increase of MDA (malondialdehyde), a biomarker of lipid damage and oxidative stress, along with a confirmed decrease in PUFA content after heavy metal exposure.

Table 2 presents the impact of toxic metals on the health risk assessment of various fish species from areas with different pollution levels. It summarizes the detected metal concentrations and evaluates potential health risks using indices such as THQ, MPI, and other risk assessment calculations.

**Table 2:** Studies that evaluated health/risk assessment of fishes from family Cyprinidae

Species	Toxic elements detected	Methods of assessment	Conclusion on consumption safety	Reference
*Chondrostoma regium* * Cyprinion macrostomus* *Barbus rajanorum mystaceus* *Capoeta trutta Carassius gibelio*	As, Cd, Pb	THQ HI CR	- THQ and HI values below 1 in every sample - Consumption was considered safe - CR value of As above acceptable level; existing carcinogenic risk for public health	(Töre et al., 2021)
*Cirrhinus reba* *Catla catla*	As, Cd, Pb	THQ TTHQ CR	- high concentration of As resulting in high THQ for As - extent THQ for Cd - *Cirrhinus reba* is considered the most toxic fish in study - consumption of these fish not fully recommended	(Alamdar et al., 2017)
*Abramis brama* *Rutilus rutilus*	As, Cd, Pb, Hg	THQ HI CR	- highest Cd concentrations observed in *rutilus rutilus* - due to high CR values, A*bramis brama* was considered potentially toxic and carcinogenic for consumption - high HI values caused mainly by As indicated health risk and fish were not recommended for human consumption	(Mielcarek et al., 2022)
*Cyprinus carpio* *Carassius gibelio* *Leuciscus aspius* *Vimba vimba* *Abramis brama*	As, Cd, Pb, Hg	THQ HI CR/TR BMF	- in the study were concentrations of toxic elements in freshwater fish being compared to seafood with conclusion of higher concentrations of Hg in freshwater fish - health/risk assessment in this study revealed no potential risk for human consumption	(Simionov et al., 2023)
*Cyprinus carpio*	Cd, Pb	BAF THQ HI CR	- all THQ and HI values below 1, resulting in no potential risk for human consumption - Cd and Pb concentrations in edible parts of the fish were below affordable limits - CR evaluation also showed non-carcinogenic risk	(Kebede & Geleta, 2023)
*Squaliobarbus curriculus*	As, Cd, Pb	MPI THQ HI CR	- MPI values showed low bioaccumulation of elements in muscle tissue compared to other tissue such as gills or liver - THQ and HI below 1 - CR values were between 1 × 10^−6^ and 1 × 10^−4^ meaning the lifetime carcinogenic risk is considered acceptable	(Jia et al., 2017)
*Carassius gibelio*	As, Cd, Pb, Hg	MPI THQ HI CR/TR	-low concentration of Pb detected -MPI for muscle tissue several times lower than for gills -although higher concentrations of As and Cd, the health/risk assessment results in no risk to human safety	(Milošković et al., 2022)
*Ctenopharyngodon idella*	As, Pb, Hg Cd (below LoD)	MPI THQ HI/TTHQ	-Pb concentrations in more than 66% samples were below LOQ (limit of quantification) -Lowest concentrations of Hg compared to other heavy metals studied -MPI, THQ and TTHQ evaluation showed no potential risks for human consumption and safety	(Kovacik et al., 2024)
*Cirrhinus mrigala* *Labeo rohita* *Catla catla*	Cd, Pb	BAF HQ (THQ) HI	- both metals were detected in muscle tissue in higher concentrations than maximum tolerable levels -highest MPI in *Cirrhinus mrigala* (0.303) - HI values > 1 -fishes from this study were considered not safe for human consumption and may pose danger for local consumers	(Junejo et al., 2019)
*Rutilus rutilus (location 1)* *Abramis brama (location 1)* *Rutilus rutilus (location 2)* *Abramis brama (location 2)*	Cd, As, Pb, Hg	Correlations	-all measured toxic metals/metalloid concentrations were below maximum allowable limits -fishes were considered safe for human consumption -negative correlation observed between Hg and some FAs -results suggest that Hg and Cd indicate lipid peroxidation, especially PUFA	(Jovičić et al., 2024)

## The influence of toxic elements on metabolism and stability of fatty acids

Toxic elements ions play a significant role in lipid peroxidation reactions and increased free radical formation. At the cellular level, these ions induce oxidative stress. Peroxidation of membrane lipids leads to alterations in their structure and properties (Chaâbane et al., 2020; Ferain et al., 2021; Fokina et al., 2013; Horton et al., 1987; Repetto et al., 2010). The lipid composition of muscle tissue in fish differ from that of mammals in the number of double bonds present. While mammalian lipids rarely contain more than two double bonds, fish lipids often include several fatty acids with five to six double bonds (Henderson, 1996; Tocher, 2003; Tocher & Glencross, 2015). These long-chain fatty acids are highly unsaturated and it is the chain length and the abundance of double bonds that make them highly vulnerable to oxidation (Das et al., 2018; Shahidi & Finley, 2001; Twining et al., 2016). In particular, valuable ω−3 fatty acids are lost as a result of peroxidation (Arab-Tehrany et al., 2012; Heshmati et al., 2019). The presence of these beneficial fatty acids in fish meat determines its quality (Mekonnen et al., 2020; Sobczak et al., 2020; Zhang et al., 2020). Peroxidation process not only disrupts metabolism but also affects the subsequent storage and processing of fish meat (German, 1999; Nie et al., 2022; Singh et al., 2022).

Lipid peroxidation results in the formation of aldehydes and ketones, known as ‘aftertaste components’, which can significantly alter the smell and taste of the fish meat (Das et al., 2018; Maqsood & Benjakul, 2011). This, of course, affects both the sensory and overall quality of the meat. The oxidation of PUFAs also forms other toxic by-products, such as acrolein, malondialdehyde, and 4-hydroxynonenal, which pose a risk to consumers (Cameron-Smith et al., 2015; Surai & Fisinin, 2010; M. Wang et al., 2022). In addition to their cytotoxic effects, some of these by-products may act as ‘anti-nutritional molecules’ and reduce the body's ability to effectively utilize the nutrients it receives (Dalleau et al., 2013; Di Nunzio et al., 2016; Maqsood & Benjakul, 2011; Pompeia et al., 2002).

**Fig. 2:**
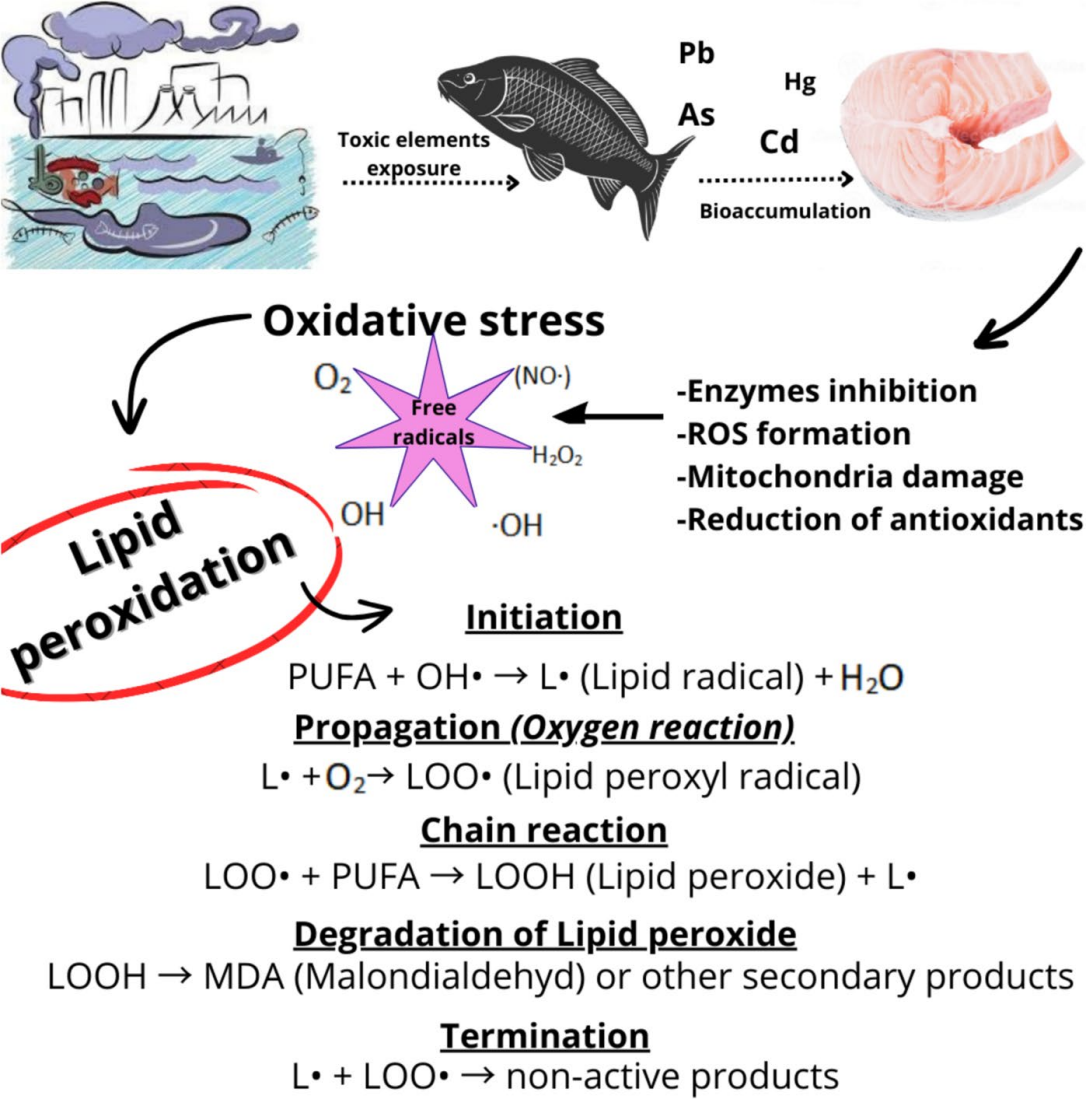
Potential effect of toxic elements on fatty acid degradation through lipid peroxidation. Scheme was created by authors using Canva (https://www.canva.com/)

Membrane phospholipids composed of PUFAs are sensitive to the effects of reactive oxygen species (Cordeiro, 2014; Elias et al., 2008; Schönfeld & Wojtczak, 2008), which are formed through interaction with environmental contaminants. Unsaturated fatty acids, whether PUFAs or MUFAs, possess structural features well suitable for reactions with metal ions (Howlett & Avery, 1997; Krznarić et al., 1983). Heavy metals such as Cd and Pb induce the formation of superoxide molecules (Eskander & Saleh, 2020; Kumar et al., 2024; Sevcikova et al., 2011). These molecules then immediately react with the double bonds of fatty acids in lipid molecules, leading to their degradation through lipid peroxidation (Das et al., 2018; Fadhlaoui & Couture, 2016). This degradation results in the destruction of their structure and decreased proportion of lipids in the tissue (Catalá, 2010; Schönfeld & Wojtczak, 2008). Figure 2 illustrates the basic mechanism of lipid peroxidation caused by heavy metal contamination in fish muscle tissue.

The short- and long-term effects of stress can lead to changes in the concentration, spectrum, and unsaturation level of fatty acids in aquatic animals, which also affect the overall metabolic precursors and products of fatty acids. As a result, the fatty acid profile of cells and tissues may be altered (Ferain et al., 2021; Fonseca et al., 2022; Javed & Usmani, 2019; Ruiz-Hernández et al., 2022). Stress-induced changes have been observed in microalgae, which are a major food source for higher aquatic animals. In this context, the PUFA synthesis capacity of freshwater algae may be negatively impacted (Chaâbane et al., 2020; B. Hussain et al., 2019). This can negatively affect upper-level organisms of the food chain in aquatic ecosystem that feed on microalgae. The nutritional composition of fish diets is known to affect the fatty acid profile of their tissues (Ghosi Mobaraki et al., 2020; Robin et al., 2003; Schulz et al., 2005). Fish are a crucial dietary source of EPA and DHA; however, they are at risk of alterations in the lipid composition of their muscle tissues, which are vital for human nutrition (Ahmed et al., 2022; Duarte et al., 2022).

An ecotoxicological study investigating the effects of different contaminants using algae as a model organism demonstrated that different contaminants cause different compositional changes in fatty acids. These changes were influenced by the type and dose of the contaminant (Duarte et al., 2022).

Another study examining the impact of toxic heavy metals, Cd, Hg and Pb on the fatty acid profiles of *Catla catla, Labeo rohita* and *Cirrhinus mrigala* caught from two polluted sites in a river revealed a decrease in the percentage of PUFAs in fish from polluted areas. This reduction resulted in losses of essential fatty acids in the fish flesh (B. Hussain et al., 2019).

A correlation has been observed between increasing environmental pollution and higher levels of SFAs in some aquatic animals. There are notable interspecies differences in the responses to selected contaminants, which are reflected in the biochemical pathways of lipid metabolism and antioxidant response to the stressor (Fonseca et al., 2022). This study also emphasized that consumers currently prefer fish from fish farms that contain lower concentrations of heavy metals.

Research by Das et al. (2018) examined the changes in fatty acid profiles of different freshwater fish species (*C. cirrhosus, C. catla, L. rohita*) caught from two differently polluted areas in India. They found that the *C. cirrhosus* species was the most affected by the presence of toxic metals (Pb, Cd). In their research, significant differences in SFA content were noted, when comparing fish from less polluted areas to those from more polluted areas. Their findings supported the theory presented in another study that exposure to bivalent metal ions in fish muscle is directly related to elevated SFA levels (Fadhlaoui & Couture, 2016). The study also confirmed a decreasing trend in PUFA and MUFA in the muscle tissue in *C. cirrhosus C. catla* with increasing concentrations of toxic metals in some species. However, in the case of *L. rohita*, findings indicated that exposure to toxic elements helped maintain PUFA levels. Specifically, eicosatetraenoic acid (C20:4n-6) and γ-linolenic acid (C18:3n-6) remained constant. These findings suggest a possible ´protective´ or alternative effect of toxic elements, particularly nickel, against fatty acid oxidative degradation, as reported in previous studies (Fadhlaoui & Couture, 2016). These findings are associated to temperature acclimation and heat stress in fish, which may have led to increased activity of enzymes such as glutathione peroxidase (GPX), catalase (CAT) and superoxide dismutase (SOD) (Forgati et al., 2017; Habashy et al., 2019; Lu et al., 2016). This enzymatic activity may help neutralize free radicals before oxidation of PUFAs occurs. However, it may not sufficiently protect metabolic pathways, mitochondrial function, homeostasis or prevent the destruction of tissues (Y. Chen et al., 2021; Onukwufor et al., 2017; Resende et al., 2022).

## Conclusions

Based on the knowledge and investigations evaluated in this review, we can conclude that the potential toxicity or hazard resulting from the consumption of Cyprinidae fish depends not only on the species of fish but also on the location where the fish are farmed or caught. The studies reviewed have reached different conclusions, but they indicate that higher exposure to the toxic elements correlates with higher risks associated with fish meat consumption.

However, clear guidelines for the consumption of these fish should be the THQ and CR calculations. If these assessments indicate a potential risk to human health, then the consumption of such fish should not be recommended, despite the beneficial fatty acids composition of the fish meat.

Our data also suggest that toxic elements affect lipid metabolism and induce lipid peroxidation, potentially leading to a reduced quality in the fatty acid profile and lowered nutritional values of the fish. Limited research has been conducted on the impact of toxic elements on the fatty acid profile, making it challenging to determine clearly whether toxic elements reduce the quality of the beneficial fatty acids in fish meat to such an extent that it would affect the present calculations to determine the risk–benefit comparisons of fish meat consumption.

On this evidence, we recommend that future research focus more on monitoring not only total toxic element exposure, but also its possible correlations with compositional changes in the fatty acid profile. We hypothesize that, if the relationships between these correlations are supported in the future, the fatty acid profile of fish muscle could emerge as an important biomarker for the determination of environmental pollution.

## Data Availability

No datasets were generated or analysed during the current study.
